# Comparison of Isotope Abundance Analysis and Accurate Mass Analysis in their Ability to Provide Elemental Formula Information

**DOI:** 10.1021/jasms.0c00419

**Published:** 2021-03-29

**Authors:** Tal Alon, Aviv Amirav

**Affiliations:** † School of Chemistry, Tel Aviv University, Tel Aviv 6997801, Israel; ‡ Afeka School of Engineering, Tel Aviv, 6910717, Israel; § Aviv Analytical Ltd., 24 Hanagar Street, Hod Hasharon, 4527713, Israel

**Keywords:** isotope abundance analysis, accurate mass analysis, elemental formula, mass spectrometry, compound identification

## Abstract

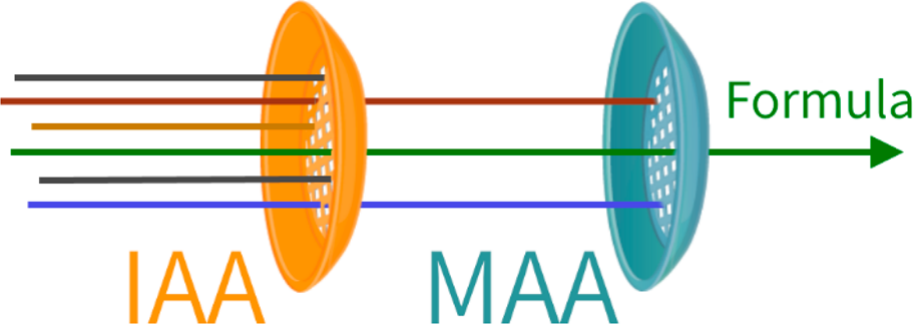

Deriving elemental formulas from mass spectra used to be an exclusive feature provided only by expensive high-resolution mass spectrometry instruments. Nowadays this feature can be used on unit resolution quadrupole-based mass spectrometers (MS) combining isotope abundance analysis (IAA) and mass accuracy analysis (MAA) with surprising accuracy that is commonly lower than 1 ppm mass accuracy. In this Article, we assess the usefulness of both MAA and IAA in the elemental formula deriving process performed on unit resolution MS data with constant resolution across the *m*/*z* range. The methods’ effective filtration power (EFP) are estimated along with their ability to provide useful elemental information under nonideal experimental conditions. The term effective mass accuracy (EMA) is introduced so that the identification power of IAA can be expressed in a familiar way and compared more readily to MAA. We found that IAA alone commonly has an EMA under 5 ppm. IAA and MAA work well together and provide improved results with median EMA < 1 ppm for calibrated MS or <3 ppm for uncalibrated MS. We have also found that even though these methods cannot be fully trusted to pinpoint the exact elemental formula under poor experimental conditions, IAA can still accurately provide the exact number of several heteroatoms such as sulfur, chlorine, and bromine, while MAA cannot. Under such conditions, a combination of both methods can also provide good insight into the amount of carbon, hydrogen, and other elements in the elemental formula.

## Introduction

Quadrupole mass spectrometers are sometimes referred to as unit-mass resolution instruments, even though it is clear that they do not deserve this title. From our experience, quadrupole mass analyzers that are used daily, without periodical mass calibration, generate mass spectral peaks that are typically 0.6–0.7 Da wide and provide centralized masses within ±0.14 Da of the actual ones. In the majority of cases, the situation is much better than that of the 26 compounds discussed in this paper that were measured with an uncalibrated GC-MS system, have an average mass error of 0.023 Da with a standard deviation (SD) of 0.011 or 88 ppm (SD = 57). These measurements were obtained with an uncalibrated GC-MS system that was mass tuned in the centroid mode over a year ago. Quadrupole-based instruments that were calibrated with PFTBA in the profile mode can be trusted to yield masses with errors within ±100 ppm, but again the majority of cases provide better mass measurements, and for seven compounds measured in this study with a calibrated instrument, the mean mass accuracy was 30 ppm (SD = 35). This surprisingly good quadrupole mass accuracy, even if much coarser than the estimation provided by the ±2 ppm mass accuracy of expensive high-resolution instruments,^1−3^ can be utilized by mass accuracy analysis (MAA) algorithms,^4−7^ listing elemental formulas with exact masses that are most similar to the measured mass, under restricted elemental range, to derive estimated elemental formulas.

The abundances of the molecular ion’s isotopologue peaks can also be analyzed using an isotope abundance analysis (IAA) algorithm, which cares for the shape of the pattern with no regard for the exact measured masses.^3,8−20^ The performance of IAA was shown to be reliable and effective at identifying compound.^3,8,17^ IAA for this paper was performed using the TAMI software.^16^

These two physically different algorithms take their data from the same source, a quadrupole-generated mass spectrum, both test the molecular ion and need it to be available, and both yield a list of elemental formulas with their match scores and identification probabilities. But how do they compare to one another? Which one has more filtration power? Which one is more trustworthy? How well do they work together?

This paper addresses these questions and provides quantitative assessments for their relative roles as well as their combination.

## Effective Filtration-Power and Mass-Accuracy

If one takes a broad look at the results provided by isotope abundance analysis or mass accuracy analysis, they both can be considered as filters. For each of these analytical methods, there is a threshold with eligible formulas on one side and irrelevant formulas on the other. The formulas that remain after filtration are the true identification candidates. We define “filtration power” (FP) as the factor by which the total number of all possible elemental formulas (with the same nominal mass, under reasonable user selected elements and criteria) is reduced via the implementation of the filter. If, for example, there are 1000 identification candidates overall, and after applying the filter, their number is reduced to 50, the filtration power is FP = 1000/50 = 20. A higher FP leads to a lower number of candidates and therefore to higher identification chances and to more trustworthy information.

We found that in a well-calibrated quadrupole GC-MS, with added PFTBA and profile (raw scan) mode scanning, we can consistently measure masses with errors under ±100 ppm, so we can trust a measured mass of ∼300 Da, for example, to be within ±30 mDa of the actual mass. We also found that an uncalibrated GC-MS (calibrated in centroid mode long ago) can still be trusted to yield masses with errors within ±140 mDa, which makes this number the minimum mass-filter window to be used when the centroid mass is reported with one point decimal precision. Note that when using the mass accuracy filters, we employ an upper mass error that is much higher than typical errors (which are about 30 ppm for a mass calibrated quadrupole) in order to make sure that the correct compounds remain in the filtered lists.

**Figure 1:**
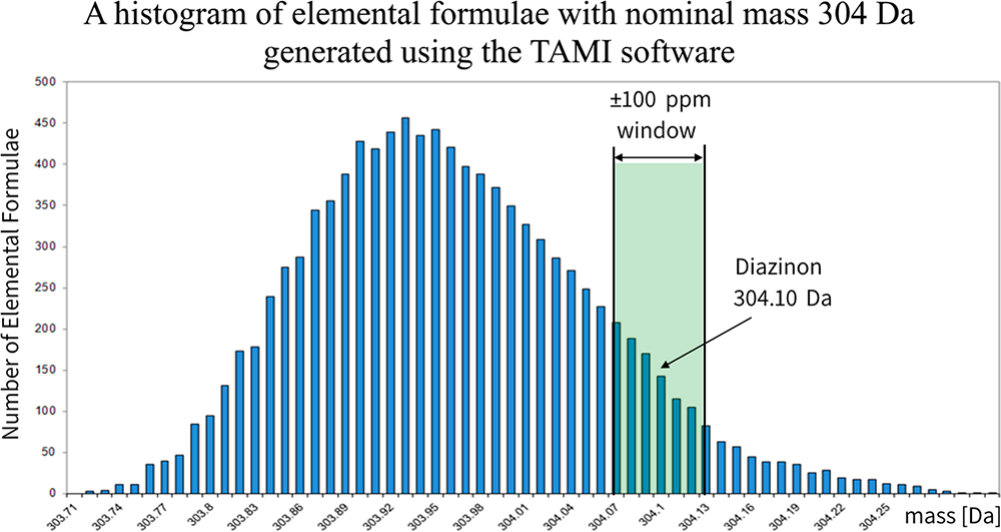
A typical histogram of elemental formulas with a nominal mass of 304 Da, obtained by the TAMI software, with no constraints on carbon and hydrogen and the following elemental ranges: O 0–8, N 0–8, S 0–4, P 0–4, Cl 0–3, Br 0–3, F 0–2. The MAA filtration window of ±100 ppm is likely to still contain many elemental formulas, resulting in a low FP. The MAA filter is much more effective for measured masses that reside on the rims of the histogram, but most measured masses do not.

Figure 1 shows the distribution of exact masses for compounds with a nominal mass of 304 Da. The shaded zone represents a filtration window with a width of ±100 ppm. One can see that most compounds are filtered out by this window. Another observation is that the performance of a mass filter is worse if the measured mass is common, while exceling around rarely seen masses at the edges of the mass defects. The distribution of exact masses of compounds with a nominal mass of 304 Da in Figure 1 was obtained with the TAMI software that provides an elemental formula from quadrupole MS data files.^16^

IAA cannot be assessed in that way, as it does not have a physical measure parameter like MAA. However, there are other ways the effectiveness of IAA and MAA can be tested and, therefore, compared by looking at the actual results they provide. Both methods produce a list of possible elemental formula, and the position of the correct formula on the list can be examined.

Table 1 compares the performance of IAA and MAA on a variety of real world compounds analyzed by a mass-calibrated GC-MS (Agilent 5977A, Agilent, Santa Clara, CA, U.S.A.) that was scanning masses in the profile mode using PFTBA at the end of the run. The actual position of the correct elemental formula in the hit list is presented, along with a new metric called effective filtration power (EFP), specifying the filtration power (ratio of all results to the results left after filtration) of the smallest filter window that would still accommodate this correct result. Effective mass accuracy (EMA) values represent the mass accuracy needed for the same filtration power. The analysis was performed in raw-scan mode (profile) with added PFTBA for the ideal mass calibration and centroiding. The results show how IAA in combination with a default ±0.14 Da MAA (can be used safely even on uncalibrated mass spectrometers with centroid data) provide great results that are equivalent or better than 3.2 ppm mass accuracy (median = 3.2 ppm) and with very high filtration power values. IAA alone with no mass filters is still very informative and provides results with a median EMA of 5.3 ppm, while MAA with a ±100 ppm window has a median EMA of 37 ppm. IAA together with a ± 100 ppm MAA combine into a very strong filter and manage to provide the correct elemental formula at the first or second hit, and commonly the first (out of hundreds or thousands of possible candidates), and the median EMA of their combination is lower than 0.3 ppm. In cases where IAA is combined with a mass filter and the result is at first place the values of EFP and EMA were calculated by, respectively, multiplying or dividing by the measured FP of the relative mass window. Nicotine, for example, is already the number one hit with IAA and no mass window, while a ±0.14 Da filter window alone leaves 113 candidates out of 241, this means 2.13 times better, so the EFP we got for the IAA is now multiplied by 2.13 and we get 514, and the EMA divided by 2.13 and we get 2.5 ppm.

**Table 1:** Showing the Performance of Isotope Abundance Analysis (IAA) and Mass Accuracy Analysis (MAA) in the GC-MS Analysis of Seven Compounds Each at 5 ng on-Column Amount, Using PFTBA Calibration in Profile Mode To Improve the Mass Measurements^a^

cmpd name, formula, and mass	No. of candidates	meas. MAA error (ppm)	IAA hit #, EFP and EMA	MAA of ±100 ppm hit #, EFP and EMA	IAA ± 0.14 Da mass filter hit #, EFP and EMA	IAA ± 100 ppm mass filter hit #, EFP and EMA
benzene, nitro-C_6_H_5_NO_2_, 123.0315 Da	78	51	1, EFP > 78, EMA < 8.3 ppm	5, FP = 16, EMA = 50 ppm	1, EFP > 78, EMA < 8.3 ppm	1, EFP > 507, EMA < 1.3 ppm
nicotine, C_10_H_14_N_2_, 162.1151 Da	241	5.1	1, EFP > 241, EMA < 5.3 ppm	2, FP = 121, EMA = 5.3 ppm	1, EFP > 514, EMA < 2.5 ppm	1, EFP > 5280, EMA < 0.2 ppm
hexadecane, C_16_H_34_, 226.2655 Da	854	37	2, EFP = 427, EMA = 198 ppm	1, FP = 854, EMA < 37 ppm	1, EFP > 17365, EMA < 4.9 ppm	1, EFP > 364658, EMA < 0.2 ppm
anthracene, C_14_H_10_, 178.0777 Da	343	24	1, EFP > 343, EMA < 4.9 ppm	6, FP = 57, EMA = 24 ppm	1, EFP > 521, EMA < 3.2 ppm	1, EFP > 4706, EMA < 0.4 ppm
caffeine, C_8_H_10_N_4_O_2_, 194.0798 Da	479	–1.4	1, EFP > 479, EMA < 0.8 ppm	2, FP = 240, EMA = 1.6 ppm	1, EFP > 755, EMA < 0.5 ppm	1, EFP > 5336, EMA < 0.1 ppm
methyl stearate, C_19_H_38_O_2_, 298.2866 Da	2398	95	7, EFP = 343, EMA = 141 ppm	5, FP = 480, EMA = 95 ppm	4, EFP = 600, EMA = 65 ppm	2, EFP = 1199, EMA = 27 ppm
chlorpromazine, C_17_H_19_ClN_2_S, 318.0952 Da	2896	73	4, EFP = 724, EMA = 1.5 ppm	138, FP = 21, EMA = 73 ppm	4, EFP = 724, EMA = 1.5 ppm	1, EFP > 4119, EMA < 0.3 ppm
descriptive statistics for EMA			avg = 51 ppm, SD = 82 ppm, med = 5.3 ppm	avg = 41 ppm, SD = 35 ppm, med = 37 ppm	avg = 12 ppm, SD = 23 ppm, med = 3.2 ppm	avg = 4.2 ppm, SD = 10 ppm, med = 0.3 ppm

a Each of the four right-most columns present the results of different analyses, IAA, MAA, IAA + MAA with a ±0.14 Da window and IAA + MAA with a ±100 ppm window. The position of the correct elemental formula in the resulting hit list is shown, and also the EFP and EMA values that are calculated as explained above. The number of candidates are all possible elemental formulae with the same nominal mass and within the following elemental range: O 0-8, N 0-5, S 0-4, P 0-2, Cl 0-2, Br 0-2 (carbon and hydrogen are not restricted). The number of isotopologue peaks used in the evaluation was 4.

The mass spectra of 26 different additional compounds were collected in the centroid mode via an Agilent 5977 GC-MS with a Cold-EI interface^21,22^ (enhances the molecular ion), which was not mass-calibrated (tuned) for over two years, and were analyzed with IAA. The results can be seen in Table 2. The most striking observation is that, for IAA, the correct compound position on the hit-list has a median value of 2. Adding a rough MAA filter of ±0.14 Da, which is the basic way the algorithm is applied when the instrument is operated in the centroid mode (typical GC-MS operation) and not mass-calibrated, brings the median position to #1 and the average EMA to 4.3 ppm (SD = 7.3) with a median of 1.3 ppm.

**Table 2:** Twenty-six Compounds, from Different GC-MS Data Files (Obtained with Cold-EI, and All with Centroid Data without Mass Calibration), Analyzed by Isotope Abundance Analysis (IAA), IAA, and Mass Accuracy Analysis (MAA) with a ±0.14 Da Window (Possible Even without Mass Calibration), and an Estimation for the Results of IAA and MAA with a ±100 ppm Window^a^

cmpd name, formula, and mass	No. of candidates	IAA result hit #, EFP, and EMA	IAA ± 0.14 Da hit #, EFP, and EMA	estimation for IAA ± 100 ppm, EFP, and EMA
dimethoate, C_5_H_12_NO_3_S_2_P, 228.9991 Da	787	#1, EFP > 787, EMA < 1 ppm	#1, EFP > 1215, EMA < 0.6 ppm	EFP > 7426, EMA < 0.11 ppm
Quinol ED, C_14_H_19_NO, 217.1461 Da	693	#2, EFP = 347, EMA = 5.1 ppm	#2, EFP = 504, EMA = 3.5 ppm	EFP = 3252, EMA = 0.54 ppm
diazinone, C_12_H_21_N_2_O_3_SP, 304.1005 Da	2413	#4, EFP = 603, EMA = 4.8 ppm	#3, EFP = 1281, EMA = 2.3 ppm	EFP = 5896, EMA = 0.49 ppm
chlorpyrifos, C_9_H_11_NO_3_Cl_3_SP, 348.9257 Da	4266	#87, EFP = 49, EMA = 9.4 ppm	#84, EFP = 174, EMA = 2.6 ppm	EFP = 699, EMA = 0.66 ppm
methidathion, C_6_H_11_N_2_O_4_PS_3_, 301.9613 Da	2456	#4, EFP = 614, EMA = 1.3 ppm	#4, EFP = 1282, EMA = 0.6 ppm	EFP = 5944, EMA = 0.13 ppm
tebuconazole, C_16_H_22_ClN_3_O, 307.1446 Da	2247	#8, EFP = 281, EMA = 5.4 ppm	#6, EFP = 584, EMA = 2.6 ppm	EFP = 2660, EMA = 0.57 ppm
iprodione, C_13_H_13_Cl_2_N_3_O_3_, 329.0328 Da	2984	#30, EFP = 99, EMA = 9.5 ppm	#28, EFP = 348, EMA = 2.7 ppm	EFP = 1479, EMA = 0.64 ppm
bifenthrin, C_23_H_22_ClF_3_O_2_, 422.1255 Da	20440	#27, EFP = 757, EMA = 1.8 ppm	#19, EFP = 2703, EMA = 0.5 ppm	EFP = 8964, EMA = 0.15 ppm
bifenazate, C_17_H_20_N_2_O_3_, 300.1468 Da	2385	#1, EFP > 2385, EMA < 0.7 ppm	#1, EFP > 5128, EMA < 0.3 ppm	EFP > 23920, EMA < 0.07 ppm
pyriproxyfen, C_20_H_19_NO_3_, 321.1359 Da	2645	#2, EFP = 1323, EMA = 1.8 ppm	#1, EFP > 4611, EMA < 0.5 ppm	EFP > 20100, EMA < 0.12 ppm
prochloraz, C_15_H_16_Cl_3_N_3_O_2_, 375.0303 Da	5447	#42, EFP = 130, EMA = 5.2 ppm	#36, EFP = 463, EMA = 1.5 ppm	EFP = 1728, EMA = 0.39 ppm
tridecane, C_13_H_28_, 184.2186 Da	421	#1, EFP > 421, EMA < 36 ppm	#1, EFP > 551, EMA < 27.5 ppm	EFP > 4191, EMA < 3.62 ppm
eicosane, C_20_H_42_, 282.3281 Da	2001	#2, EFP = 1001, EMA = 29 ppm	#1, EFP > 2077, EMA < 14 ppm	EFP > 10297, EMA < 2.82 ppm
triacontane, C_30_H_62_, 422.4846 Da	6575	#13, EFP = 506, EMA = 123 ppm	#3, EFP = 1804, EMA = 34.5 ppm	EFP = 5976, EMA = 10.41 ppm
HMTD, C_6_H_12_N_2_O_6_, 208.069 Da	648	#1, EFP > 648, EMA < 0.6 ppm	#1, EFP > 954, EMA < 0.4 ppm	EFP > 6421, EMA < 0.06 ppm
xylazine, C_12_H_16_N_2_S, 220.1029 Da	841	#2, EFP = 421, EMA = 2.3 ppm	#1, EFP > 654, EMA < 1.5 ppm	EFP > 4159, EMA < 0.23 ppm
phenothiazine, C_12_H_9_NS, 199.045 Da	444	#1, EFP > 444, EMA < 0.5 ppm	#1, EFP > 617, EMA < 0.4 ppm	EFP > 4339, EMA < 0.05 ppm
propranolol, C_16_H_21_NO_2_, 259.1567 Da	1183	#1, EFP > 1183, EMA < 1.1 ppm	#1, EFP > 2105, EMA < 0.6 ppm	EFP > 11372, EMA < 0.11 ppm
triflupromazine, C_18_H_19_F_3_N_2_S, 352.1216 Da	11410	#1, EFP > 11410, EMA < 0.01 ppm	#1, EFP > 40619, EMA < 0.01 ppm	EFP > 161497, EMA < 0.01 ppm
promethazine, C_17_H_20_N_2_S, 284.1342 Da	1992	#4, EFP = 498, EMA = 4.1 ppm	#4, EFP = 1051, EMA = 1.9 ppm	EFP = 5178, EMA = 0.39 ppm
promazine, C_17_H_20_N_2_S, 284.1342 Da	1992	#2, EFP = 996, EMA = 1.4 ppm	#2, EFP = 2102, EMA = 0.7 ppm	EFP = 10355, EMA = 0.13 ppm
chlorpromazine, C_17_H_19_ClN_2_S, 318.0952 Da	2896	#1, EFP > 2896, EMA < 0.3 ppm	#1, EFP > 6349, EMA < 0.1 ppm	EFP > 27944, EMA < 0.03 ppm
haloperidol, C_21_H_23_ClFNO_2_, 375.1396 Da	10955	#38, EFP = 288, EMA = 3.6 ppm	#30, EFP = 1028, EMA = 1 ppm	EFP = 3835, EMA = 0.27 ppm
benzene, propyl-, C_9_H_12_, 120.0934 Da	90	#1, EFP > 90, EMA < 2.9 ppm	#1, EFP > 59, EMA < 4.4 ppm	EFP > 693, EMA < 0.38 ppm
propane, 1,3-dibromo-, C_3_H_6_Br_2_, 199.8831 Da	574	#1, EFP > 574, EMA < 7.8 ppm	#1, EFP > 801, EMA < 5.6 ppm	EFP > 5607, EMA < 0.8 ppm
finasteride, C_23_H_36_N_2_O_2_, 372.2771 Da	4553	#1, EFP > 4553, EMA < 3.4 ppm	#1, EFP > 16183, EMA < 1 ppm	EFP > 60860, EMA < 0.25 ppm
descriptive statistics for EMA		avg = 10.1 ppm	avg = 4.3 ppm	avg = 0.9 ppm
		SD = 24.5 ppm	SD = 8.4 ppm	SD = 2.1 ppm
		median = 3.2 ppm	median = 1.3 ppm	median = 0.3 ppm

a The results for #1 hits in IAA and MAA columns are calculated using the estimated filtration power of the mass window (the ratio of the full mass defect range divided by 100 ppm). The number of candidates are all possible elemental formulae with the same nominal mass and within the following elemental range: O 0-8, N 0-5, S 0-4, P 0-2, Cl 0-2, Br 0-2 (Carbon and Hydrogen are not restricted), except for the following changes: for Chlorpyrifos Cl 0-4, for Bifenthrin F 0-3, for Prochloraz Cl 0-4, for Triflupromazin F 0-3, and for Haloperidol F 0-2. The number of isotopologue peaks used was usually 4, and it was raised when the measured pattern exhibited information in higher masses (multiple chlorine/bromine for example).

The benefit of a mass window added to IAA analysis is estimated, in this table, by factoring in the change in mass-range. For example, in the analysis of dimethoate, there are 787 candidates that span a mass defect range of ±943.57 ppm, so we estimated that a mass filter of ±100 ppm will result in a filtration factor of around 9.44 for the combined IAA and EMA (relative to IAA).

The IAA combination with ±100 ppm MAA is therefore estimated to yield an average EMA of 0.9 ppm (SD = 2.1) and a median of 0.3 ppm. This means that the IAA + MAA combination commonly performs better than expensive high resolution instrument and is approximately equivalent to 1 ppm mass accuracy that is operated with no consideration for isotope abundances.

Together with the previous table data, IAA has a median EMA of 3.6 ppm, IAA combined with MAA of ±0.14 Da has median EMA = 1.5 ppm and IAA with MAA of ±100 ppm has median EMA = 0.3 ppm. 85% of all IAA ± 100 ppm MAA analyses shown in this paper show an equivalency to a ±1 ppm accurate mass instrument.

Note that the higher the mass is, the more combinations of elements are expected to be considered (not always, but usually).^23^ As the number of combinations grow, a perfect filtration algorithm, working on perfect data will make sure the correct formula is always identified (number 1 on the list) and will therefore get higher and higher EFP. This was not something we saw in our experiments, and it is probably due to imperfect data, as noise has a huge effect, especially on isotopologues low in abundance.

## Conclusive Information under Uncertain Conditions

The reason one applies IAA or MAA to mass spectral data is to acquire the elemental formula of measured compounds. One question is how can such tools be trusted and to what extent.

Any elemental formula deriving process results in an ordered list of possible elemental formulas with declining matching to the experimental data and identification probabilities. When applying the right analysis settings and if the data is clear and not corrupted by excessive noise, poor ion statistics, offsets or other skewing effects, the first elemental formula on the list has a decent chance of being the correct one. Nonideal or poor experimental conditions obviously reduce the possibility of this sought after first place identification, but even under the best conditions, the correct formula can appear somewhere down the list, as there are usually many candidates with similar characteristics. This identification uncertainty means that for true unknowns we must always consider not only the first elemental formula on the list, but also a portion of those that come after it.

We found that when IAA is used, the group of best-matching formulas exhibits high similarities in terms of the number of heteroatoms such as chlorine, bromine, and sulfur, since these elements have highly distinct isotopologue patterns. Accordingly, IAA provides an incredibly reliable heteroatom identification method, which is not obtained with mass accuracy alone, by relying on the characteristics of an entire group of good results. Using IAA on the measured mass-spectrum of diazinon (C_12_H_21_N_2_O_3_PS), for example, with the TAMI software^16^ elemental formula generator, outputs many elemental formula candidates out of which all eligible candidates (error < 0.1, a TAMI software metric which is a function of the various distances of the various abundances in the isotopologue pattern) have exactly 1 sulfur atom and no chlorine or bromine atoms, making these numbers fully certain (under the following elemental constraints: O 0–8, N 0–5, S 0–4, P 0–2, Cl 0–2, Br 0–2). This means that, regardless of the elemental formula found in the first place by the algorithm, one can assert with confidence that the measured compound has no chlorine or bromine and exactly one sulfur atom.

The results from MAA, within the ±100 ppm threshold we consider as reliable, give no such information, as the number of chlorine, bromine, and sulfur atoms (and the other elements tracked) widely varies among this best matching group.

This high uncertainty of MAA, encountered in mass-analysis, is caused in part by the inherently low average filtration power of the method (as previously discussed), but there is another intrinsic problem that hinders its performance: the reliance on only one feature, the measured mass of the monoisotopic peak. This feature is not specific enough, as there are many possible elemental combinations that yield similar masses.

A short glance at Table 3, which shows the 10 best matching compounds in terms of mass, all within 5 ppm error compared to diazinon, makes the problem obvious. One can see that these best mass-matching compounds have a widely varying elemental composition. An oxygen atom together with a phosphorus atom, for example, have almost the exact same mass as a carbon atom and chlorine, making the mass of the second elemental formula different by only 1.8 × 10^–4^ Da from that of diazinon.

**Table 3:** Ten Elemental Formula Most Mass-Similar to Diazinon (#1)^a^

#	elemental comp.	mass error (ppm)
1	C_12_H_21_N_2_O_3_S_1_P_1_	0
2	C_13_H_21_N_2_O_2_Cl_1_S_1_	0.582044
3	C_14_H_18_N_4_P_2_	–1.249587
4	C_20_H_17_O_1_P_1_	2.144028
5	C_21_H_17_Cl_1_	2.726072
6	C_10_H_16_N_4_O_7_	2.791840
7	C_22_H_12_N_2_	–3.291675
8	C_15_H_22_O_2_Cl_2_	–4.485359
9	C_13_H_23_N_2_Cl_1_P_2_	4.768161
10	C_14_H_22_O_3_Cl_1_P_1_	–5.067404

a Showing the wildly varying number of elements present. Most formulas do not include sulfur or phosphorous (which are present in the correct formula), and some include chlorine (not present). The range of carbon atom numbers is 10–22.

IAA, on the other hand, uses several features together: the various isotopologue ions present in the spectrum. Table 4 shows the 10 best matching compounds in terms of IAA error, and as can be seen, all of them have much in common. Some elements like chlorine, bromine, sulfur, selenium, and silicon have unique effects on the relative abundances of the isotopologue ions that are rarely missed by the IAA algorithm.

**Table 4:** Ten Elemental Formula Most Similar in Their Isotopologue Pattern (IAA) to Diazinon (#1)^a^

#	elemental comp.	IAA error (%)
1	C_12_H_21_N_2_O_3_S_1_P_1_	0
2	C_13_H_6_O_3_S_1_P_2_	0.003
3	C_12_H_5_N_2_O_4_S_1_P_1_	0.006
4	C_12_H_6_N_2_O_2_S_1_P_2_	0.011
5	C_13_H_22_O_2_S_1_P_2_	0.011
6	C_11_H_20_N_4_O_4_S_1_	0.011
7	C_11_H_21_N_4_O_2_S_1_P_1_	0.019
8	C_13_H_21_O_4_S_1_P_1_	0.019
9	C_11_H_5_N_4_O_3_S_1_P_1_	0.021
10	C_12_H_22_N_2_O_1_S_1_P_2_	0.022

a All show one sulfur atom, none shows chlorine atoms, most show phosphorous, and the number of carbon atoms is 11–13. Comparing to the wildly varying MAA results shown in Table 2, these results are much more cohesive.

Table 5 shows some further specific examples: The elemental spread provided by IAA, MAA, and their combination from the analysis of diazinon, anthracene, caffeine, cholesterol, chlorpromazine, and dibromopropane. In order to see the negative effect brought on by the reliance on only one feature, only the 20 best results were taken into account. This eliminated the difference in Filtration power between the two methods (without this step, MAA would have shown far worse results than IAA). As can be seen, IAA provides clearer results with less uncertainty, and the combination of both methods provides the best results.

**Table 5:** Elemental Spread within the 20 Best Results, Analyzing the Mass Spectrum of Diazinon, Caffeine, Anthracene, Cholesterol, Chlorpromazine, and Dibromopropane Using IAA, MAA, and Their Combination with 100 ppm Mass Accuracy^a^

Diazinon								
	C	H	O	N	S	P	Cl	Br
actual	12	21	3	2	1	1	0	0
IAA	11–13	4–25	1–6	0–4	*1*	0–2	*0*	*0*
MAA	8–22	12–24	0–8	0–4	0–3	0–2	0–2	0–1
IAA + MAA	10–14	16–26	1–6	0–4	*1*	0–2	*0*	*0*
Caffeine								
actual	8	10	2	4	0	0	0	0
IAA	8–10	7–12	0–3	0–4	*0*	0–2	*0*	*0*
MAA	5–14	10–19	0–6	0–4	0–2	0–2	0–1	*0*
IAA + MAA	8–9	10–11	2–3	2–4	*0*	0–1	*0*	*0*
Anthracene								
actual	14	10	0	0	0	0	0	0
IAA	12–14	6–22	0–1	0–2	*0*	*0*	*0*	*0*
MAA	4–14	10–18	0–5	0–4	0–2	0–2	0–1	*0*
IAA + MAA	*14*	*10*	*0*	*0*	*0*	*0*	*0*	*0*
Cholesterol								
actual	27	46	1	0	0	0	0	0
IAA	26–28	2–46	0–3	0–4	*0*	0–1	*0*	*0*
MAA	21–27	42–51	0–4	0–4	0–1	0–1	0–1	*0*
IAA + MAA	21–28	42–51	0–4	0–4	*0*	0–2	*0*	*0*
Chlorpromazine								
actual	17	19	0	2	1	0	1	0
IAA	15–19	0–31	0–2	0–4	*1*	0–1	*1*	*0*
MAA	7–19	14–26	0–8	0–4	0–2	0–2	0–2	0–1
IAA + MAA	13–21	11–27	0–4	0–4	0–2	0–1	*1*	*0*
Dibromopropane								
actual	3	6	0	0	0	0	0	2
IAA	1–3	2–6	0–1	0–2	*0*	*0*	*0*	*2*
MAA	0–6	0–6	0–4	0–4	0–4	0–2	0–2	0–2
IAA + MAA	*3*	*6*	*0*	*0*	*0*	*0*	*0*	*2*

a Correct identifications are italic. IAA provides a narrower spread for most elements and therefore greater certainty in the amounts of each of them. The combination yields superior results with smaller uncertainties.

## Discussion and Conclusions

When analyzing mass spectral data, IAA and MAA are both useful techniques, each utilizing a different physical attribute of the molecular ion. In terms of identification, IAA as it is commonly used with a wide ±0.14 Da mass window provides the correct elemental formula most of the time (median position on the resulting hit-list is #1), and is equivalent to mass analysis with under 3 ppm mass accuracy. When IAA is coupled with MAA using a ± 100 ppm window (on mass-calibrated quadrupoles operated in profile mode), the performance surpasses that of existing high resolution instruments and provides an EMA of under 1 ppm.

IAA was also found to provide more reliable elemental information. The correct number of chlorine, bromine, and sulfur atoms can be found in all eligible results provided by the IAA algorithm, making their determination a near certainty, whereas MAA results vary drastically in this aspect, even when one considers only a few of the best results. The IAA + MAA combination can further reduce the uncertainty about the number of atoms of the elements.

This strong determination of certain heteroatoms favorably affects the determination of other more common elements, as it naturally introduces a strong restriction that greatly reduces the number of possible options (if some heteroatoms are surely present, it leaves a smaller mass range to accommodate other elements into).

It should be noted that both MAA and IAA require that the molecular ion will be present, and IAA unlike MAA relies on the measurement of low abundance isotopologues and thus suffer from statistical fluctuations approximately 100× more than MAA alone and thus typically require a few nanograms on-column sample amounts or using a second run with a narrow mass spectral window for improved ion statistics. The problem of a weak or missing molecular ion can be overcome via the use of GC-MS with Cold EI that provides significantly enhanced molecular ions^21,22^ yet with full compatibility with NIST library identification.

GC-MS based sample identification usually begins with a NIST library^24^ (or another library) search and identification, which provides the sample name and structure and often includes isomer level differentiation, which cannot be deducted from an obtained elemental formula. Therefore, library-based identification is the best tool, when applicable. Unfortunately, the majority of compounds are not included in any library, and in these cases, obtaining the sample elemental formula is the best way for sample characterization. The TAMI software uses IAA to automatically check the library results and alert the user if they seem erroneous (usually since the compound is not recorded in the library) and, in those cases, provide an IAA + MAA alternative, yielding the most probable elemental formulas.

In conclusion, when attempting to obtain elemental formulas with quadrupole MS based data files, it is highly effective to use IAA as the main algorithm, with a coarse ±0.14 Da mass accuracy filter, when standard centroid files are analyzed, or ±100 ppm if the MS is calibrated in the profile mode. This combination is very likely to help in the determination of the elemental formula in general and to accurately determine the presence and number of various heteroatoms even under nonideal experimental conditions. Combining these two algorithms with a library search algorithm (such as NIST’s^24^), when it is applicable, yields a very comprehensive solution to compound identification via quadrupole based mass spectrometry.

The abundances of the various isotopes used by the TAMI software are those recommended by the Commission on Isotopic Abundances and Atomic Weights (CIAAW) of the International Union of Pure and Applied Chemistry (IUPAC) on 2009.^25^
